# Major depressive disorder as a risk factor for suicidal ideation for attendees of educational institutions: a meta-analysis and meta-regression

**DOI:** 10.1590/1984-0462/2023/41/2021344

**Published:** 2023-03-13

**Authors:** Laura Costa Souza, Lucas Pequeno Galvão, Henrique Soares Paiva, Cintia de Azevedo-Marques Périco, Antonio Ventriglio, Julio Torales, João Maurício Castaldelli-Maia, Anderson Sousa Martins-da-Silva

**Affiliations:** a Secretaria de Saúde de São Bernardo do Campo, São Bernardo do Campo, SP, Brazil.; b University of Foggia, Foggia, Italy.; c National University of Asunción, School of Medical Sciences, San Lorenzo, Paraguay.; d Universidade de São Paulo, São Paulo, SP, Brazil.

**Keywords:** Suicidal ideation, Adolescent, Youth, Major depressive disorder, Ideação suicida, Adolescente, Juventude, Transtorno depressivo maior

## Abstract

**Objective:**

This study aimed to analyze the effect of major depressive disorder (MDD) as a risk factor for suicidal ideation in individuals whose ages varied from 11 to 24 years and who were attending educational institutions.

**Data source:**

A systematic review was carried out by searching in PubMed and Biblioteca Virtual em Saúde (BVS). Original studies conducted in educational institutions, including individuals whose age varied from 11 to 24 years, in English, Spanish, or Portuguese were included.

**Data synthesis:**

Eight studies were selected for the meta-analysis, including 35,443 youths, with an average age of 16.8 years, predominantly female (51.2%), and from middle-income Asian countries (91.6%). An odds ratio of MDD of 3.89 (95%CI 2.46–6.17) for suicide ideation in youth was found. Subgroup analysis showed higher effects in Asia (OR=4.71; 95%CI 3.22–6.89) than Americas (OR=1.71; 95%CI 1.44–2.03). The meta-regression model indicated that younger adolescents (coef=-0.63; 95%CI 1.09–-0.18; p<0.01) and older studies (coef=-0.23; 95%CI 0.039–-0.08; p<0.01) presented higher effects of MDD on suicidal ideation.

**Conclusions:**

Early detection and treatment of MDD in youth patients are of utmost importance for preventing suicidal ideation. Educational institutions could play an important role in the early detection and intervention.

## Resumo

**Objetivo:**

Analisar o efeito do transtorno depressivo maior (TDM) como fator de risco para a ideação suicida em indivíduos com idades entre 11 e 24 anos que frequentam instituições de ensino.

**Fontes de dados:**

Revisão sistemática realizada até agosto de 2020 por meio de buscas nas bases United States National Library of Medicine (PubMed) e Biblioteca Virtual em Saúde (BVS), sem limitações quanto à data de publicação.

**Síntese dos dados:**

Oito estudos foram selecionados para a metanálise, incluindo 35.443 jovens com idade média de 16,8 anos, predominantemente do sexo feminino (51,2%) e de países asiáticos de renda média (91,6%). Foi encontrado *odds ratio* (OR) de TDM de 3,89 (intervalo de confiança — IC95% 2,46–6,17) para a ideação suicida em jovens. A análise de subgrupo mostrou efeitos maiores na Ásia (OR=4,71; IC95% 3,22–6,89) do que nas Américas (OR=1,71; IC95% 1,44–2,03). O modelo de metarregressão demonstrou que adolescentes mais jovens (coef=-0,63; IC95% -1,09–-0,18; p＜0,01) e estudos mais antigos (coef=-0,23; IC95% -0,039–-0,08; p＜0,01) apresentaram maiores efeitos do TDM sobre a ideação suicida.

**Conclusões:**

Observou-se relação entre TDM e ideação suicida no continente asiático, entre adolescentes mais jovens e em estudos mais antigos.

## INTRODUCTION

Suicide is a complex and multifaceted phenomenon with a multifactorial etiology. There are many factors involved in suicidal behavior, including adverse experiences in early life, genetic and cultural characteristics, and traumatic experiences.^1^ Suicidal ideation usually precedes suicide attempts and consummated suicide, encompasses characteristics such as self-destructive thoughts, and wishes plans possessed by individuals who have the purpose of ending their lives.^2^ About 80% of the cases of suicide present a mental disorder, with major depressive disorder (MDD) being the most common.^3^

Despite being a complex phenomenon, suicide can be prevented, especially with early interventions such as treatment and prevention of mental disorders, awareness of suicide and mental health, and limited access to means.^4^

The vulnerability of adolescents for suicide behavior was shown in a study,^5^ including individuals aged between 12 and 18 years, in which 19.8, 12.8, and 8.7% reported unstructured suicidal ideation, structured suicidal ideation, and attempted suicide at least once in the past 12 months, respectively.

MDD is considered a consistent predictor of suicidal behaviors in children and teenagers, as confirmed in multiple studies from different countries. In South Korea, Juon et al.^6^ reported that among 10–21 years old students, those with severe MDD were 5.3 times more likely to report suicidal ideas and 3.2 times more likely to attempt suicide when compared to students with mild MDD. In both Hong Kongese (average age of 15.7 years old) and American (average age of 15.9 years old) students, MDD predisposed to suicidal ideation.^7^

Self-injurious behaviors (SIBs) are common in adolescence. Brazil, for example, had an increase of 39.8% in the notifications from 2018 to 2019, and the age group from 15 to 19 years concentrated 23.3% of the cases.^1^ There has been some debate about SIB being an independent diagnosis or a risk factor for a suicide attempt, ideation, or consummation. In 2016, a meta-analysis appointed SIB as a risk for later suicidal thoughts and behaviors.^8^

The main purpose of this meta-analysis was to analyze the effect of MDD as a risk factor for suicidal ideation in individuals whose age varied from 11 to 24 years, who were attending educational institutions.

## METHOD

The first step review of the literature included all original studies conducted in educational institutions (schools and colleges) with no restriction regarding the country of origin, including individuals whose age varied from 11 to 24 years, investigating MDD effects on suicidal ideation. Other studies aimed to investigate MDD as a risk factor for suicidal behaviors, suicide attempts, and consummated suicide, regardless of the age of the participants, were excluded, as well as studies in languages other than English, Spanish, and Portuguese, non-original articles.

The search was conducted in two electronic databases: PubMed database of the U.S. National Library of Medicine and BVS (initials in Portuguese that stand for “Biblioteca Virtual em Saúde”). The last search date was August 2020. We failed to find any similar ongoing meta-analysis in PROSPERO. Our systematic review protocol was registered on Open Science Framework with the following link: https://osf.io/sznpv.

In Pubmed, we employed the following Medical Subject Headings (MeSH), through the following search strategy: (((“Suicide”[Mesh]) AND “Risk Factors”[Mesh]) AND “Adolescent”[Mesh]) AND “Self-Injurious Behavior”[Mesh]. In BVS, we employed the following Health Science Descriptors (DeCS: initials in Portuguese that stands for *Descritores em Ciência da Saúde*), using the following search strategy: (tw:(suicídio)) AND (tw:(fatores de risco)) AND (tw:(*adolescentes*)) AND (tw:(*automutilação*)). In Scielo, we searched through DeCS, using the following search strategy: (*suicídio*) AND (*fatores de risco*) AND (*adolescentes*) AND (*automutilação*). In these searches, “self-injurious behavior” and *automutilação* were also considered synonyms of suicide attempts.

**Figure 1.**
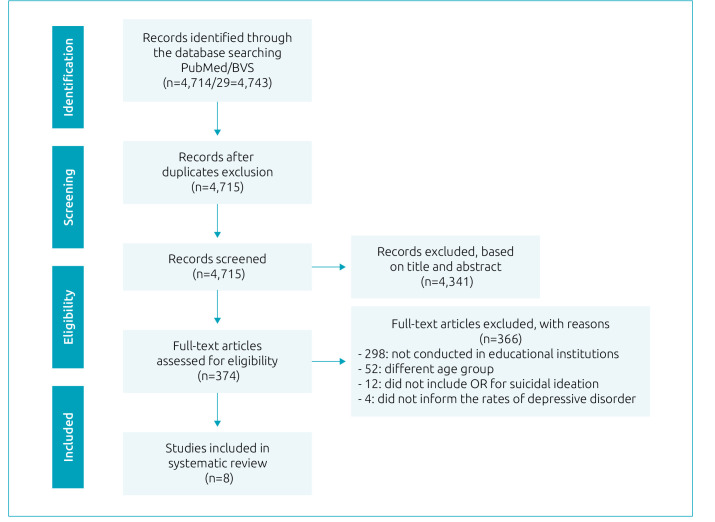
Flow diagram of study selection.^9^

Figure 1 shows the study selection, following the PRISMA statement.^9^ The first and the last author conducted the selection process independently as the first step. We found 4,743 studies: 4,714 in PubMed, 29 in BVS. After excluding duplicates, 4,715 studies were considered primarily through titles and abstracts, and 4,341 studies did not meet the inclusion criteria. Afterward, the remaining 374 studies were fully read, and the other 366 were excluded for not meeting the inclusion criteria.

The first and last authors read all the eight studies included independently. Then, the first author tabulated the data. Then, the tabulated data were evaluated by the last author. In cases of discordance between the first and last authors on the information to be presented in the review, the penultimate author made the decision on the best way to report the data. The included articles were all evaluated by the JBI Critical Appraisal Checklist for Studies Reporting Prevalence Data to address the possibility of bias.

This review reported information on the following variables: title of the study, author(s), year of publication, type of the study, sample size, methodology, sample characteristics (e.g., gender, average age, assessment scales, continents, and countries in which the studies were conducted), study design, outcome measures, and odds ratio (and 95%CI) for MDD as a risk factor for suicidal ideation.

We included the logOR (and their 95%CI) of the eight studies in a meta-analysis model and used a random-effects model because a high heterogeneity was expected. The logOR was calculated since the log is necessary to calculate the confidence interval because of the asymmetrical nature of the OR scale^10^. I^2^ has been calculated as a measure of between-study heterogeneity. Data were analyzed employing STATA software version 16. Then, we carried out a meta-regression model for gender, age, year of publication, and continents. Forest plots of the cumulative meta-analysis were derived for the first three continuous variables. Finally, we conducted a subgroup meta-analysis for the continent of the study, which was a categorical variable.

## RESULTS

**Figure 2.**
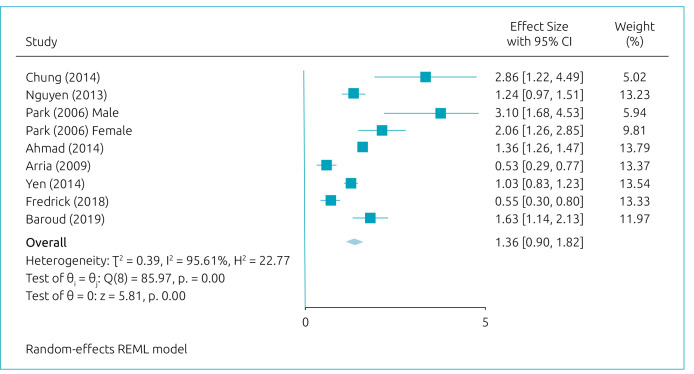
Meta-analysis: estimated MDD logOR for suicidal ideation.^11-18^

Tables 1-3 present the main information from the eight studies included. Seven are observational cross-sectional, and one is a longitudinal cohort study. Sample sizes varied from 403 to 25,174 (median=1,249; IQR=583–2,241). All studies included females and males^11-18^.Females represented most of the sample in six studies.^11-16^ The average proportion (males:females) was 0.95. The average age was 16.8 years (range=11–24). Most of the studies have been conducted in Asia (n=6),^11-13,15,17,18^ followed by North America (n=2).^14,16^ Various scales were used to evaluate suicidal ideation (e.g., MINI-Kid [Mini International Neuropsychiatric Interview- Kid], BDI [Beck Depression Inventory], K-SADS-E [The Kiddie Schedule for Affective Disorders and Schizophrenia Epidemiological version for School-Age Children], SIQ-JR [the Suicidal Ideation Questionnaire-Junior], and DAWBA [Development and Well-Being Assessment]), and MDD (MINI-Kid, CES-D Scale [Center for Epidemiological Studies Depression], DASS21 [The Depression, Anxiety and Stress Scale – 21 Items], BDI, MC-CES-D [Mandarin Chinese version of the Center for Epidemiological Studies – Depression Scale], CDI 2 – short version [The Children’s Depression Inventory 2], and DAWBA). Three scales (i.e., MINI-Kid, BDI, and DAWBA) evaluated both suicidal ideation and MDD.^14,17,18^ The estimated MDD logOR for suicidal ideation was somewhat significant (logOR=1.36; 95%CI=0.90–1.82/OR=3.89; 95%CI 2.46–6.17) (Figure 2).^11-18^

**Table 1.** Depression as a risk factor for suicidal ideation among adolescents in educational institutions: data extraction from included studies.

Author, year	Study population	Study design	Outcome measures (caseness cutoff)	Odds ratio (95%CI)
Park et al.^12^ (2006)	n: 1,312 Mean age: NA Gender: 654M:658F (0.993) Nationality: Asia (South Korea) Education levels: grades 10–12	Observational cross-sectional	Definition: depressive disorders; suicidal ideation Instrument(s): - Depressive disorders: CES-D Scale - Suicidal ideation: Suicidal ideation was measured as a single-item measure (“Have you ever seriously considered attempting suicide during the past 2 weeks?” yes/no)	Males: 22.25 (5.35–92.49) Females: 7.81 (3.53–17.28)
Arria et al.^14^ (2009)	n: 1,249 Mean age: 17–19 years Gender: 605M:644F (0.939) Nationality: North America (USA) Education levels: grades 11–12	Longitudinal cohort study	Definition: depressive disorders; suicidal ideation Instrument(s): - Depressive disorders: BDI - Suicidal ideation: BDI (Item 9 of the BDI pertains to suicidal thoughts and was recoded into a binary variable to denote the presence or absence of suicide ideation in the past few days)	1.70 (1.30–2.10)

NA: not applicable; CES-D Scale: Centre for Epidemiology Studies Depression Scale; BDI: Beck Depression Inventory.

**Table 2.** Depression as a risk factor for suicidal ideation among adolescents in educational institutions: data from included studies.

Author, year	Study population	Study design	Outcome measures (caseness cut-off)	Odds ratio (95%CI)
Nguyen et al.^11^ (2013)	n: 1,161 Mean age: 16.1 (15–19) years Gender: 424M:737F (0.575) Nationality: Asia (Vietnam) Education levels: Ggrades 10–12	Observational cross-sectional	Definition: Depressive disorders; suicidal ideation Instrument(s): - Depressive disorders: CES-D Scale - Suicidal ideation: Questions about ever having seriously considered suicide and the making of a suicide plan, employed of a scale ranging from never, sometimes, and often. A yes/no question was also used to identify students who had attempted suicide	3.45 (2.63–4.54)
Chung et al.^17^ (2014)	n: 607 Mean age: 13.1 (SD=1.4) years Gender: 305M:302F (1.009) Nationality: Asia (Taiwan) Education levels: grades 5–9	Observational cross-sectional	Definition: Depressive disorders; suicidal ideation Instrument(s): - Depressive disorders: MINI-Kid (≥5) - Suicidal ideation: MINI-Kid (≥1)	17.40 (3.40–89.50)
Ahmad et al.^13^ (2014)	n: 25,174 Mean age: 12–17 years Gender: 12487M:12687F (0.984) Nationality: Asia (Malaysia) Education levels: grades 7–11	Observational cross-sectional	Definition: Depressive disorders; suicidal ideation Instrument(s): - Depressive disorders: DASS21 - Suicidal ideation: Suicidal ideation was defined as “ever seriously considered attempting suicide in the past 12 months”	3.91 (3.53–4.33)

MINI-Kid: Mini International Neuropsychiatric Interview-Kid; CES-: Centre for Epidemiology Studies Depression Scale; DASS21: Depression Anxiety and Stress Scale.

**Table 3.** Depression as a risk factor for suicidal ideation among adolescents in educational institutions: data from included studies.

Author, year	Study population	Study design	Outcome measures (caseness cutoff)	Odds ratio (95%CI)
Yen et al.^15^ (2014)	n: 5027 Mean age: 15–24 years Gender: 2317M:2710F (0.854) Nationality: Asia (Taiwan) Education levels: gades 7–12	Observational cross-sectional	Definition: Depressive Disorders; Suicidal Ideation Instrument(s): - Depressive Disorders: MC-CES-D - Suicidal Ideation: K-SADS-E	2.80 (2.30–3.40)
Fredrick et al.^16^ (2018)	n: 403 Mean age: 13–16 years Gender: 199M:203F (0.980) Nationality: North America (USA) Education levels: grade 9	Observational cross-sectional	Definition: Depressive Disorders; Suicidal Ideation Instrument(s): - Depressive Disorders: CDI 2 Short Version - Suicidal Ideation: SIQ-JR	1.74 (1.31–2.16)
Baroud et al.^18^ (2019)	n: 510 Mean age: 11–17 years Gender: 284M:226F (1.256) Nationality: Asia (Lebanon) Education levels: Elementary or middle school, secondary school, university	Observational cross-sectional dataset	Definition: Depressive Disorders; Suicidal Ideation Instrument(s): - Depressive Disorders: DAWBA - Suicidal Ideation: DAWBA	5.12 (3.13–8.38)

K-SADS-E: Epidemiological version of the Kiddie-Schedule for Affective Disorders and Schizophrenia; MC-CES-D: Mandarin Chinese Version of the Centre for Epidemiological Studies-Depression Scale; CDI 2 Short Version: Children’s Depression Inventory 2nd Edition Short Version; SIQ-JR: Suicidal Ideation Questionnaire-Junior Version; DAWBA: Development and Well-Being Assessment.

**Figure 3.**
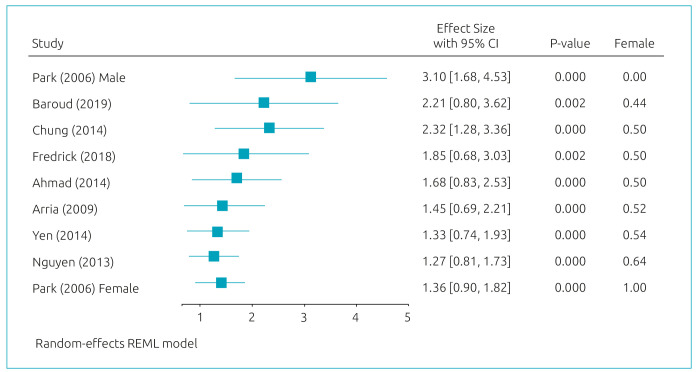
Cumulative Meta-analysis: Gender effects.

**Figure 4.**
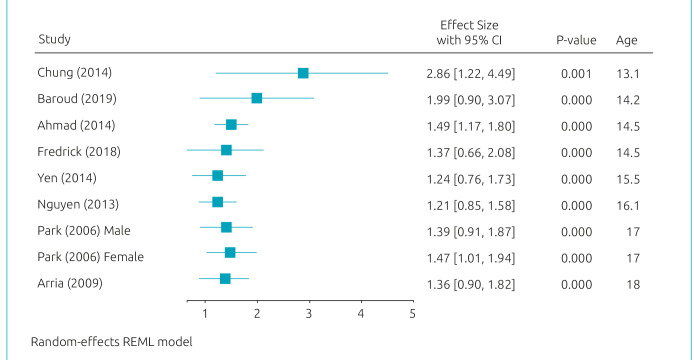
Cumulative Meta-analysis: Age.

**Figure 5.**
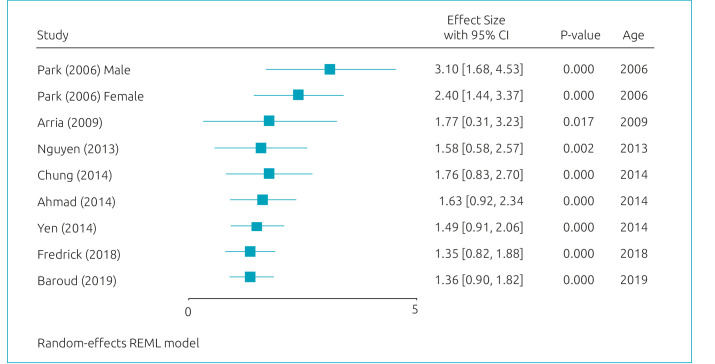
Cumulative Meta-analysis: Year of Publication.

Figure 2 presents the results of the meta-regression model. Age, year of publication, and continent have shown significant effects on the relationship between MDD and suicidal ideation. Figures 3-5 present cumulative meta-analyses for gender, age, and year of publication, respectively. In Figures 4 and 5, the earlier the age and year of publication, the higher the MDD logOR for suicidal ideation. We also performed a subgroup meta-analysis for continents, in which Asia presented a significantly larger logOR (logOR=1.55; 95%CI 1.17–1.93/OR=4.71; 95%CI 3.22–6.89) than Americas and (logOR=0.54; 95%CI 0.37–0.71/OR=1.71; 95%CI 1.44–2.03). 

## DISCUSSION

The present review gathered articles investigating MDD as a risk factor for suicidal ideation. Suicidal ideation is associated with an elevated risk for suicide attempts, representing an imminent risk of suicide, especially among adolescents.^19^ The association between MDD and suicidal ideation is well known by any mental health professional. However, this was the first meta-analysis, to the best of our knowledge, reporting on the ORs for this association among adolescents in education institutions.

We found a logOR of 1.36 in the present meta-analysis. These findings may be compared to a broader meta-analysis conducted by Guzmán et al.^20^ investigating psychopathology-related variables predicting suicidal thoughts and behaviors. They found that the experience of suicidal thoughts and behaviors increased with the presence of general psychopathology. Mental disorders were well-known risk factors for suicide, being present in up to 90% of youths that commit suicide.^21-23^

The incidence of suicidal ideation is higher among old than young adolescents.^24^ In the U.S. National Comorbidity Survey, the incidence was lower than 1% and approximately 17% for those 10 and 18 years old, respectively.^24^ However, our meta-regression showed an inverse pattern regarding age. We found that the younger the age of the individual, the greater the association between MDD and suicidal ideation. However, the lower mean age in the present meta-analysis was 13.1 years old. 

Previous studies also found a relationship between gender, MDD, and suicidal ideation.^25,26^ This finding was not supported by our study. The meta-regression did not show a significant relationship between gender and suicidal ideation.

In unfavorable socioeconomical contexts, adolescents show higher rates of suicidal attempts than those who live in a favorable socioeconomical context.^27,28^ This evidence may lead countries with lower socioeconomic levels to take juvenile suicide as a priority in their public health policy.^29,30^ In 2016, 80% of global suicides occurred in middle- and low-income countries,^31^ with a relevant number of adolescents in their population.^32^ Another study^33^ has shown that one over five teenagers in low-income countries, aged 13–17 years, reported death thoughts, suicidal ideation, or had attempted suicide in the past 12 months (16.9, 17, and 17%, respectively). These prevalence rates were higher when compared to those of middle- and high-income countries. Our meta-regression (Table 4) confirmed these findings, since the ORs found in Asia and North America were 0.55 (1.17–1.93) and 0.54 (0.37–0.71), respectively, with a tight correlation between MDD and suicidal ideation in low- and middle-income countries when compared to high-income ones. In our report (Tables 1-3), out of the eight selected studies, five^11,13,15,17,18^ were conducted in middle-income countries (Taiwan, Vietnam, Malaysia, and Lebanon) and three^12,14,16^ were conducted in high-income countries (the United States and South Korea).

**Table 4.** Meta-regression of age, gender, year of publication, and continents.

	Coefficient	Standard error	Z-score	p＞|z|	95% confidence interval	
Age	-0.64	0.23	-2.74	0.006	-1.10	-0.18
Female	-0.83	1.02	-0.81	0.416	-2.83	1.17
Year	-0.24	0.08	-2.98	0.003	-0.40	-0.08
Continents	493.5	164.8	2.99	0.003	170.4	816.6

Residual heterogeneity: tau^2^=0.2183; I2 (%)=89.22; H^2^=9.28; R^2^ (%)=44.71; Wald chi^2^(3)=9.31; prob＞chi^2^=0.0255. Test of residual homogeneity: Q_res=chi^2^(5)=30.49; prob＞Q_res=0.0000.

Finally, we found a significant trend in the year of publication, with older studies reporting a higher association between MDD and suicidal ideation. This evidence might be explained considering the lack of policies, globally, regarding the children-youth mental health services and suicide prevention in the past decades. Recently, an increasing number of children and adolescent’s mental health services, specific medical training, health promotion, and prevention policies have been promoted in many countries.^34,35^

We opted to assess suicidal ideation attending educational institutions since suicide prevention strategies for this age group seem to be more effective in this setting when compared to traditional interventions,^36^ as demonstrated by Wasserman et al.^37^

Regarding the limitations of the study, the first is that our meta-analysis included studies conducted with students at school or college only. The second limitation includes that the answers provided by adolescents might have been affected by some cultural and social factors, including fear or shame, and provided in a socially expected manner. Finally, the third limitation includes the predominance of Asian and middle-income countries in the selected articles for this meta-analysis, which may influence the comparisons between middle-/low-income countries and high-income countries.

## CONCLUSION

The present review found that MDD is a relevant risk factor for suicidal ideation among youths from 11 to 24 years old in educational institutions. In addition, younger age, older date of publication as well as the country of origin (Asian country) were associated factors with a tighter correlation between MDD and suicidal ideation. Thus, early detection of MDD and early interventions among adolescents are encouraged. Moreover, educational institutions may also play a relevant role in the early MDD detection programs.^38,39,40^
